# ROS responsive polyethylenimine-based fluorinated polymers for enhanced transfection efficiency and lower cytotoxicity

**DOI:** 10.17305/bjbms.2021.6704

**Published:** 2022-05-01

**Authors:** Peng Hua, Donglin Yang, Ruie Chen, Peiqi Qiu, Meiwan Chen

**Affiliations:** State Key Laboratory of Quality Research in Chinese Medicine, Institute of Chinese Medical Sciences, University of Macau, Macau, China

**Keywords:** Fluorination, ROS-responsive, serum-resistant, polycation, transfection

## Abstract

Cationic polymer polyethylenimine (PEI) plays a crucial role in gene delivery. However, high molecular weight PEI leads to higher efficient transfection efficacy and higher cytotoxicity, while low molecular weight PEI exhibits lower transfection performance with lower toxicity. Therefore, effective chemical modification of PEI is required to enhance transfection activity and improve biocompatibility. Here, reactive oxygen species (ROS) responsive PEI-based fluorinated polymers (TKPF) with three degrees of fluorination (TKPF 12.5%, TKPF 25%, and TKPF 50%) were designed and synthesized by crosslinking low molecular weight PEI (PEI 1.8K) with a thioketal (TK) linker and then modifying heptafluorobutyric anhydride onto their surface. Fluorination reduced the positive charge density and endowed hydrophobic and lipophobic characteristics to resist serum interactions. The fluorophilic effect mediated efficient cellular uptake and endosomal escape while ROS-responsive TK linker allowed the polyplex disassembly to decrease the cytotoxicity of the polycations and improve the release of payloads at specific sites. TKPFs attained superior transfection efficiency in multiple cell lines (293TN cells and B16F10 cells) *in vitro* and showed excellent biocompatibility. TKPFs also exhibited great serum resistance in gene delivery and TKPF 50% transfected nearly 80% cells in the presence of 70% FBS. These results demonstrate that the fluorination and ROS responsiveness combined polycations are excellent gene-delivery vectors with serum-resistant capacity for further application.

## INTRODUCTION

Gene therapy has shown great prospects for conquering human diseases by introducing exogenously produced genes in cells to express functional proteins [1-3]. Numerous genetic targets and therapeutic nucleic acids (e.g., plasmid DNA, antisense oligonucleotides, microRNA, short hairpin RNA, and small interfering RNA) have endowed gene therapy with broad applications in various diseases such as monogenic diseases, neurodegenerative disorders, chronic diseases, infectious diseases and cancer [4-8]. The current key issue for effective gene therapy *in vivo* is to construct efficient and safe gene vectors because the therapeutic efficacy of naked genes is limited by low stability and poor cellular uptake caused by the presence of nuclease and negatively charged biological cell surfaces [9,10]. Viruses have evolved to deliver the genetic material into host cells, which are used clinically as gene vectors with relatively high transfection efficiency. However, the inevitable immunogenicity, pathogenicity and time-consuming preparation procedures limit their further application [11]. Non-viral vectors including cationic amphiphiles, cationic polymers and dendrimers have been deemed as safer and more efficient alternatives for gene delivery [12]. Among them, cationic polymers represented by polyethyleneimine (PEI), polyamidoamine (PAMAM), poly-2-dimethylaminoethyl methacrylate (PDMAEMA), poly (b-amino ester) (PBAE), and chitosan are gradually occupying the mainstream due to the diversity in chemical structure, ease of modifications, and low immunogenicity [13-19]. There are drawbacks hindering effective and safe gene transfection *in vivo* due to the potential high toxicity and poor delivery capacity across multiple physiological barriers. Therefore, further modification of cationic polymers is required for constructing novel vectors with increased transfection efficiency and decreased toxicity [20,21].

Fluorination and introducing biodegradable bonds have emerged as two practical strategies to improve the transfection efficiency and reduce the toxicity of gene carriers. On one hand, fluorination modification endows polycation carriers with good serum stability because fluorinated chains are hydrophobic and lipophobic, which can resist disruptive action of lipid-containing serum components as compared to conventional lipids [22]. In addition, the fluorophilic effect has been reported to enhance cellular uptake and endosomal escape by facilitating polycations to transport across the cellular lipid bilayer [23,24]. These characteristics inspired us to synthesize fluorinated polycations to efficiently enhance the performance of cationic gene carriers. On the other hand, introducing biodegradable bonds in crosslinked low molecular weight polycations to replace high molecular weight polycations is a potential approach to overcome the high toxicity of cationic polymers [25,26]. Endogenous stimuli involving low pH, abundant reductive glutathione (GSH) in the cytoplasm, hypoxia, and high reactive oxygen species (ROS) level have been widely utilized to develop safe and efficient bio-responsive gene carriers. Such biodegradable crosslinked polymers can be used to promote intracellular disassembly and improve intracellular bioavailability with enhanced transfection efficacy and decreased cytotoxicity of polycations. Hence, combining fluorination and biodegradable sensitive bonds is an ideal strategy to develop such gene vectors. Only a few studies have reported and investigated the transfection efficiency as well as the mechanisms including polyplexes formation, cytotoxicity, protein adsorption, cellular uptake, and lysosome escape of these systems.

In this study, a series of ROS responsive PEI-based fluorinated polymers (TKPFs) were synthesized by crosslinking low molecular weight PEI (MW=1.8K) with a thioketal (TK) linker to obtain a high molecular weight polymer (TKPEI), followed by modifying TKPEI with heptafluorobutyric anhydride with the grafting ratio of 0, 12.5%, 25%, and 50%. Considering the inert property of fluorous compounds, the transfection efficiency of TKPFs was investigated in the presence of high serum contents (70%). Moreover, the investigation of gene condensation capacity, protein absorption, cellar uptake efficiency, and lysosome escape ability further accounted for the superiority of TKPF 50%, which facilitated to reveal the structure-activity relationship of gene vectors and the role of fluorination modification in transfection. It also provided insights into the gene vector design.

## MATERIALS AND METHODS

### Materials

Branched polyethyleneimine 1.8 KDa (PEI 1.8K), N-(3-dimethylaminopropyl)-N’-ethylcarbodiimide hydrochloride (EDCI), N-hydroxysuccinimide (NHS), heptafluorobutyric anhydride, chlorpromazine (CPZ), methyl-beta-cyclodextrin (m-βCD), and genistein (GNT) were purchased from Aladdin Reagents (Shanghai, China). Amiloride (AMI) was obtained from Macklin (Shanghai, China). Branched polyethyleneimine 25 KDa (PEI 25K) and chloroquine were purchased from Sigma-Aldrich (St. Louis, MO, USA). Enhanced green fluorescent protein plasmid DNA (pcDNA3.1-EGFP, CMV promoter, 6119 bp) was obtained from OBiO Technology (Shanghai, China). Endotoxin-free plasmid DNA isolation kit was bought from Tiangen Biotech Co., Ltd. (Beijing, China). Hoechest3342, lysotracker-green, ammonium persulfate (APS), and 5x protein loading buffer were procured from Beyotime Biotechnology (Shanghai, China). Dexamethasone was purchased from Dalian Meilun Biology Technology Co., Ltd (Liaoning, China).

### Cell culture

Human embryonic kidney 293TN cell line (HEK-293TN) and murine melanoma B16F10 cell line used as model cells for gene transfection were purchased from American Type Culture Collection (ATCC, Rockville, MD, USA) and cultured in Institute of Chinese Medical Sciences (Macau, China). These cell lines were cultured with Dulbecco’s modified Eagle’s medium (DMEM, Gibco, USA) supplemented with 10% (v/v) fetal bovine serum (FBS, Gibco, USA), 100 units per mL penicillin, and 100 mg/mL streptomycin (Gibco, USA) in 5% CO2 atmosphere at 37°C.

### Synthesis of TKPEI and TKPF

The synthesis of thioketal (TK) was based according to previously reported literature [27]. 5.2 g of 3-mercaptopropionic acid and 5.8 g of anhydrous acetone were briefly mixed and stirred at room temperature under a saturated hydrogen chloride atmosphere for 6 hours. Then the reactant was sealed in an ice-salt mixture to condense until complete crystallization. Then, the obtained crystal was filtered and washed with n-hexane and ice water. Next, the purified product (TK) was collected after vacuum drying. The attained TK was then crosslinked with PEI 1.8K to synthesize TKPEI. 0.37 mmol of EDCI and 0.37 mmol of NHS were added in 0.3 mmol of TK-dissolved anhydrous methanol. After stirring for 2 hours at room temperature, 0.28 mmol of PEI 1.8K dissolved in 4 mL of anhydrous methanol was added to the mixture with stirring at room temperature to react overnight. The obtained product was dialyzed with dialysis pouch (MWCO: 2000 Da, Viskase, USA) in ultrapure water for 3 days, and the external water was changed 3 times a day. Finally, the solution was freeze-dried to yield a transparent, slightly viscous gel-like solid. For TKPF synthesis, 0.2 mmol of TKPEI was dissolved in 1 mL of anhydrous methanol and different amount of TK linker (0.025 mmol, 0.05 mmol, and 0.1 mmol) were added to the solution with 350 mL of triethylamine used as the deacidification agent and stirred for 24 hours. Subsequently, the mixture was dialyzed and lyophilized to obtain TKPF with different fluorination ratio (12.5%, 25%, and 50%). The structural characterization of the synthesized TKPF was further analyzed by ^1^H-NMR.

### Preparation and characterization of TKPEI/pDNA and TKPF/pDNA polyplexes

TKPEI, TKPF 12.5%, TKPF 25%, TKPF 50%, PEI1.8K, and PEI25K (1 mg/mL aqueous solution) were mixed with EGFP plasmid DNA at different mass ratios for 0.5 hours to form polyplex. The particle size and zeta potential of TKPF/pDNA polyplexes were determined by Zetasizer Nano ZS 90 (Malvern, UK).

### Agarose gel electrophoresis assay

The nucleic acid condensation ability of TKPFs was evaluated by gel electrophoresis assay. Transfection polyplexes consisting of different polymers and pDNA were prepared at different weight ratios (ranging from 0.5:1 to 40:1). Gel electrophoresis was performed in 5X DNA loading buffer at 150V for 15 min. The gel electrophoresis image was observed by the Gel-Doc System (Bio-Rad, USA).

### Cytotoxicity evaluation

The cytotoxicity of a series of TKPFs was determined by MTT assays on 293TN cells. Cells were seeded at a density of 5000 cells per well in a 96-well plate and were cultured overnight. After the cells grew to an appropriate density, the medium was replaced by fresh medium supplemented with different concentrations of TKPF (3.125-50 mg/mL). After 48 hours, culture medium was replaced by MTT-containing medium (1 mg/mL) for extra 4 hours of incubation. Then, medium was removed, followed by the addition of 100 mL of DMSO in each well. Then absorbance at 570 nm was measured by a microplate reader (BioTek, USA).

### *In vitro* gene transfection efficiency

pEGFP was used as the reporter gene to evaluate in vitro transfection efficiency. 293TN or B16F10 cells were seeded in a 24-well plate with DMEM medium and incubated at 37°C overnight. When cells increased to 70-80% confluency, the medium was replaced by serum-free medium for 1 hour starvation. The transfection polyplexes were constructed by mixing 50 μL of EGFP plasmid (0.5 μg in serum-free medium) with 50 μL of TKPF, and then the prepared transfection polyplexes were added to the cell culture plate in proportion and incubated for 0.5 h. At the same time, PEI1.8K and PEI25K were used as control gene vectors for transfection. After 6 hours of culture, cells were incubated for additional 42 hours with fresh medium containing 10% FBS. The expression level of green fluorescence protein was qualified by an inverted fluorescence microscope (Leica, Germany) and quantified by a flow cytometer (BD, USA). The evaluation of serum-resistant transfection performance of TKPFs was conducted by replacing the serum-free medium with serum-containing medium at different concentrations of FBS (0%, 10%, 30%, 50%, 70%, and 90%).

### SDS-PAGE analysis

Transfection polyplexes containing pDNA and different polymers were dissolved in 100 μL of PBS and then 900 μL of FBS/PBS mixed solution with different proportions of FBS was added to mimic blood with high salt and high serum. After incubating at 37°C for 30 min, the samples were loaded and ran on the gel for about 1-2 h. The gel was then immersed and stained by Coomassie brilliant blue at 37°C for 1 h, and decolorized (methanol: acetic acid: water = 40: 40: 20) for 30 min. The obtained protein bands were observed by taking photos.

### Cellular uptake and mechanism exploration

HEK-293TN cells were seeded in 96-well plates with 8000 cells per well and attached overnight. Then pEGFP labeled with cy5 was encapsulated in PEI25K NPs, TKPEI NPs and TKPF NPs for transfection. NPs were added into each well and 6 hours later cells were washed with PBS for invert fluorescence microscopy (Leica, Germany) observation and collected for flow cytometry. To further investigate the uptake mechanism, cells were pre-treated with cellular uptake inhibitor including GNT (350 μM), CPZ (20 μM), m-βCD (5 μM), AMI (100 μM) for 1 h before transfection.

### Lysosomal escape detection

Gene polyplexes consisting of polymers and cy5-labelled DNA were prepared and transfected as mentioned in cellular uptake study. After 4 hours or 6 hours of transfection, the treated cells were washed with cold PBS twice and stained with lysotracker green for 1 h. Then the cells were washed with PBS twice and the nuclei of cells were stained by Hoechst 33324 for 30 min. Cells were finally fixed with 4% paraformaldehyde and observed by DMI8 inverted fluorescent microscope (Leica, Germany).

### Statistical analysis

All experiments were performed 3 times. The data were analyzed by OriginPro^®^ 9.1 (Northampton, MA, USA) and presented as the mean ± standard deviation (SD). The significant difference was tested by one-way analysis of variance (p < 0.05 were considered statistically significant; *represented as p < 0.05, **as p < 0.01 and ***as p < 0.001).

## RESULTS

### Synthesis and characterization TKPEI and TKPF

**FIGURE 1:**
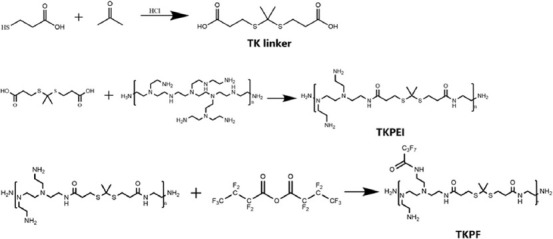
Synthetic route and chemical structure of TKPEI and TKPF.

**FIGURE 2:**
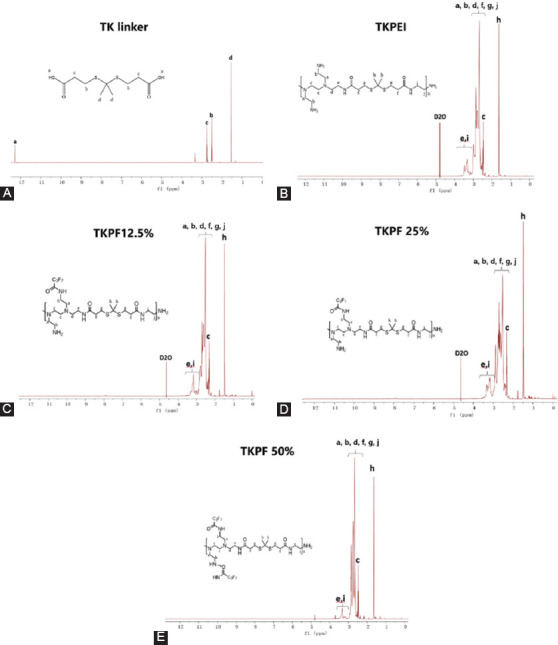
The 1H NMR spectrum of (A) TK, (B) TKPEI, (C) TKPF 12.5%, (D) TKPF 25%, and (E) TKPF 50% in D_2_O.

Crosslinking low molecular weight PEI to form a high molecular weight gene vector is one of the effective methods used to solve the weight-dependent dilemma about the transfection efficiency and toxicity of PEI [28]. The TK linker with degradability ability in oxidative environment was used as a connecting unit to crosslink PEI 1.8K. The obtained biocompatible gene carrier was easy to be degraded and released pDNA into the cell. The synthesis procedure of TKPEI and TKPF was illustrated in Figure 1. The successful synthesis of TK linker was confirmed by 1H NMR spectrum, which showed typical peaks of TK linker such as hydroxyl hydrogen at both ends (δ = 12.28), methyl hydrogen in the middle part (δ = 1.51) and ethyl hydrogen (δ = 2.51, 2.74) (Figure 2A). After crosslinking TK linker with PEI 1.8K, the spectra of TKPEI (Figure 2B) revealed a series of ethyl hydrogen in the structure of PEI and a characteristic methyl peak (H peak, δ = 1.51) in the TK linker. The hydroxyl hydrogen peak of TK linker disappeared in the structure of TKPEI, indicating the successful crosslinking of TKPEI due to the substitution of the active hydroxyl site. TKPEI was further fluorinated to synthesize TKPFs with different fluorination degrees. Since the fluorinated group was at the distal end of the polymer and was far from the hydrogen atom, it did not cause a significant effect on the chemical environment of the hydrogen atom. Additionally, the chemical shift of it was similar to that of TKPEI in 1H NMR spectra (Figure 2C-E).

### Formation and properties of polycation/DNA polyplexes

**FIGURE 3:**
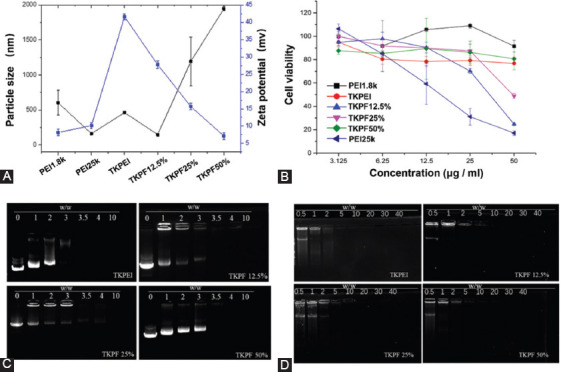
Characterization and cytotoxicity evaluation of TKPEI/pDNA and TKPFs/pDNA polyplexes. (A) Size and zeta potential; (B) Cell viability on 293TN cells; (C-D) The DNA encapsulation capability of TKPEI and TKPFs at various w/w ratios evaluated by gel electrophoresis.

**FIGURE S1:**
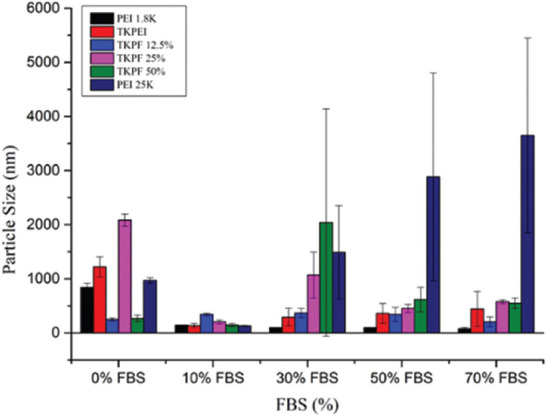
Particle size of the TKPF/pDNA polyplexes in medium containing different concentrations of FBS.

TKPEI NPs and TKPF NPs binary nanocomplexes were fabricated by mixing pDNA with TKPEI and TKPF respectively. The properties such as particle size and zeta potential of the polyplexes were subsequently determined by DLS. The results of Figure 3A revealed that TKPEI and TKPF 12.5% formed nanocomplexes with hydrodynamic diameter about 200-400 nm while the particle sizes of the TKPF 25% and TKPF 50% complexes were irregular at the transfection weight ratio (30:1). The large particle sizes of TKPF 25% and TKPF 50% NPs might be attributed to their neutral zeta-potential, which resulted in the minimized repulsion and triggered aggregation. The zeta potential of TKPEI is shown in Figure 3A was determined as 41.6 ± 0.7 mV. Fluorination of TKPEI significantly reduced the positive charge. With the increase of fluorination ratio, the zeta potential of TKPF 12.5%, TKPF 25% and TKPF 50% decreased to 27.8 ± 0.9, 15.7 ± 0.8, and 7.1 ± 0.7 mV, respectively. And the polyplexes formed by TKPEI and TKPFs showed a smaller particle size when adding 10% FBS, which means the synthesized carriers were more stable in the serum (Figure S1).

The gene loading capacity of the polymers was investigated by using agarose gel electrophoresis. As shown in Figure 3C-D, each polycation tightly wrapped the DNA at the transfection mass ratio (30:1), indicating their good DNA condensation capabilities. TKPEI fully encapsulated compressed nucleic acid at a relatively low weight ratio (<3:1) due to the strongest positive charge density. TKPFs with higher fluorination degrees required slightly higher dosage for full retardation, which might be attributed to the reduction of positive charge density caused by fluorine atom. Overall, the four synthesized gene vectors can effectively compress nucleic acid at around 3:1 with slight difference.

### Cytotoxicity

Safety is the prerequisite for gene delivery carrier. Here, we evaluated whether biodegradable bonds and fluorination decreases the cytotoxicity of polycation. As shown in Figure 3B, the cytotoxicity of the synthesized TKPEI and TKPFs was higher than that of PEI1.8K and lower than that of PEI25K overall. This indicates that the simple crosslinking method with bio-responsive linker succeeded in overcoming the molecule weight-dependent toxicity because the crosslinked TKPEI remained low cytotoxicity in a wide concentration range from 3.125 to 50 μg/mL. Specifically, the viability of cells treated by TKPF 50% in the transfection concentration (23-25 μg/mL) was up to 90%. Even at a relatively high concentration (50 μg/mL) of TKPF 50%, the cell viability was kept above 80%, exhibiting excellent biocompatibility. However, high concentration of TKPF 12.5% or TKPF 25% (50 μg/mL) exhibited sharply increased toxicity, revealing that low degree of fluorinated modification might cause toxicity to some extent. For TKPF 50%, more fluorine atoms were present in the structure, which reduced the charge density of the material, thus leading to lower cytotoxicity than TKPF 12.5% or TKPF 25%. Therefore, crosslinking low molecule weight PEI with degradable bonds and fluorinated cationic polymers are believed to reduce cytotoxicity.

### *In vitro* transfection efficiency

**FIGURE 4:**
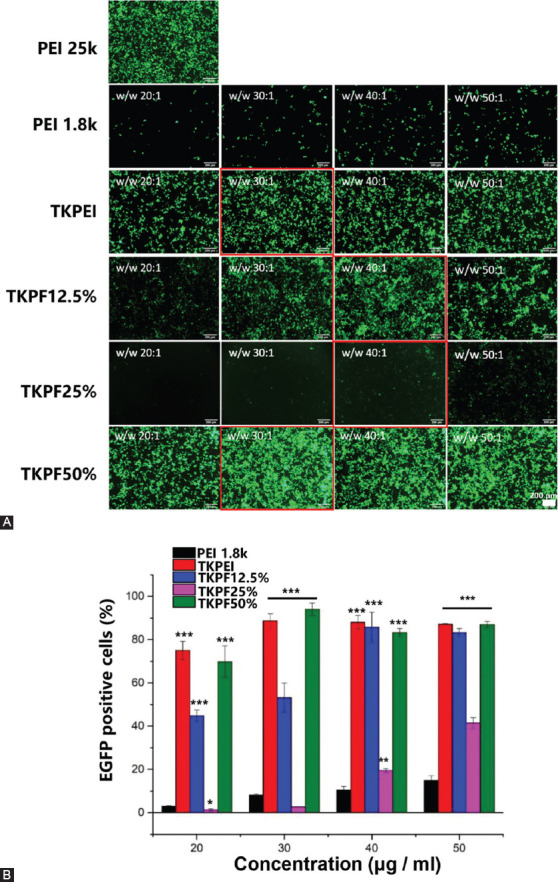
293TN cells transfected by various plasmid-loaded polyplexes. (A) Images of EGFP expression at various w/w ratios taken by fluorescence microscopy; (B) EGFP positive cells transfected by polyplexes of TKPEI and TKPFs at different w/w ratios. PEI25K with the ratio of 2:1 was used as control. (Scale bar: 200 µm).

The transfection performances of TKPEI and TKPFs were investigated in 293TN and B16F10 cells under serum-free environment. The results in Figure 4A showed that TKPEI and TKPF 50% exhibited excellent transfection efficiency in 293TN cells at an optimal N/P ratio (30:1 for TKPEI and 40:1 for TKPF 50%), which was even superior to the “gold standard” PEI25K at optimal w/w ratio of 2:1. In addition, the fluorination method did not always result in higher transfection efficiency considering TKPF 50% transfected 293TN cell with high transfection efficiency (> 95%) and high fluorescence intensity in a single cell while TKPF 12.5% and TKPF 25% did not attain satisfied transfection results (Figure 4B). Hence, the number of fluorine atoms obviously affected the transfection efficiency and better transfection results might be obtained for TKPFs with more fluorine atoms present in the structure.

**FIGURE S2:**
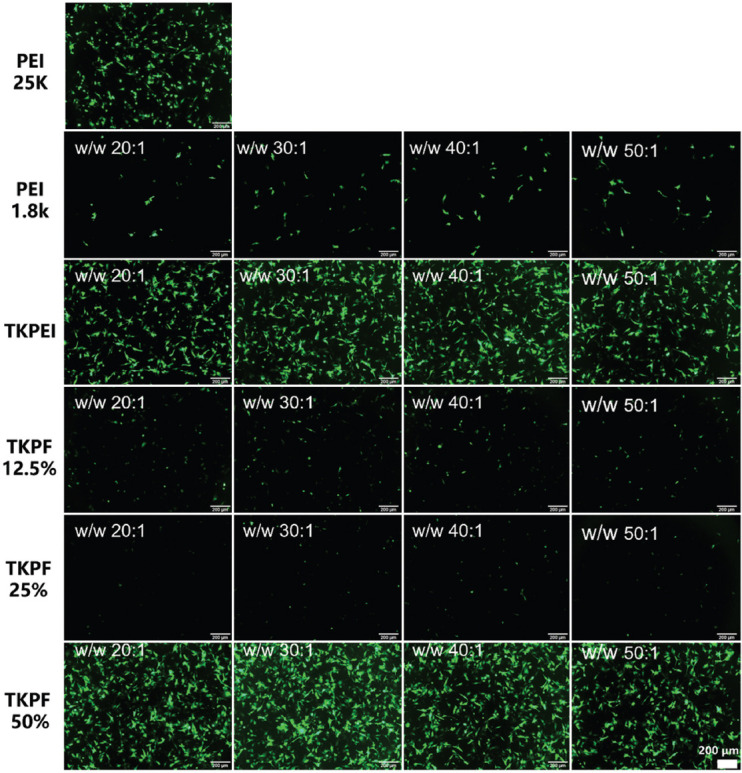
Fluorescent images of EGFP expression in B16F10 cells at various w/w ratios. PEI 25 kDa was used as control. (Scale bar: 200 μm).

For B16F10 cells, the transfection performance of each vector was similar to that of 293TN cells (Figure S2). Both TKPEI and TKPF50% effectively transfected B16 cells, and the transfection efficiency of TKPF50% was more superior than PEI25K. These merits might be attributed to the higher level of intracellular ROS in B16F10 cells, where TK linker was easily cleaved in response to ROS. Therefore, the cleavage of TKPEI and TKPF50% quickly released the encapsulated genes and further improved the gene transfection, showing their therapeutic potential for tumor treatment.

### Evaluation of serum-resistant transfection performance

**FIGURE 5:**
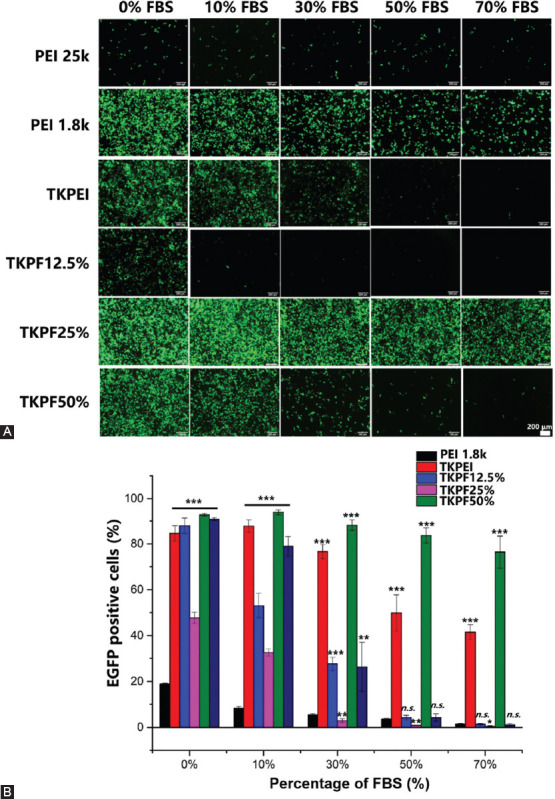
293TN cells transfected by various plasmid-loading polyplexes in DMEM medium containing different concentrations of FBS. (A) Images of EGFP expression taken by fluorescence microscopy; (B) EGFP positive cells transfected by polyplexes of TKPEI and TKPFs. (Scale bar: 200 µm).

It is well known that the transfection efficiency sharply decreases when polycation-based gene carriers are exposed to serum [29]. Therefore, the essential requirement for *in vivo* gene vector is to develop a stable gene carrier in blood circulation with a lot of serum. To investigate whether the synthesized gene vectors could maintain the transfection performance in serum-containing medium, pEGFP-encapsulated polyplexes in DMEM with FBS content ranging from 0% to 90% were added to the cells to assess the serum resistant property of these polyplexes for gene therapy. As shown in Figure 5A, the transfection efficiency of PEI25K was excellent in the absence of serum, but it decreased drastically once serum was added. The transfection efficiency of PEI25K dropped to about 20% in DMEM with 30% FBS, and sharply decreased to nearly zero with FBS proportion increasing to 50%, showing typically low serum tolerance effect. Surprisingly, TKPF 50% showed potent serum-resistant transfection performance, which still transfected nearly 80% cells in medium with 70% FBS. In addition, the green fluorescence intensity of a single cell was very high, indicating that TKPF 50% effectively resisted the serum absorption and other interferences. The empowered good serum-resistant stability of TKPF 50% might be attributed to the anti-fouling feature of fluorine atoms to avoid protein absorption and maintain structural integrity. TKPEI also maintained moderate transfection ability in DMEM containing high level of FBS, indicating that the stability of gene vectors in serum can be achieved simply by cross-linking low molecular weight PEI. The exact mechanisms might be connected with the rigid structure of TKPEI, which could have facilitated vectors to be stable in serum.

TKPEI and TKPF 50% exhibited good serum-resistant transfection property (Figure 5B), showing great potential in gene therapy *in vivo*. Although fluorination modification resulted in extremely superior serum-resistance for TKPF 50%, other two fluorinated polymers, TKPF 12.5% and TKPF 25% did not achieve the desired effect. TKPF 12.5% and TKPF 25% performed worse than non-fluorinated TKPEI, meaning that only specific fluorination ratio could benefit serum resistance. Therefore, we then investigated the behavior of each gene vector on multiple steps of transfection including protein absorption, cellular uptake, and lysosome escape to further uncover the composite effect of the fluorination and influence of FBS on transfection. With this, we hope to provide references for the design of vectors for *in vivo* gene therapy.

### Protein absorption

**FIGURE 6:**
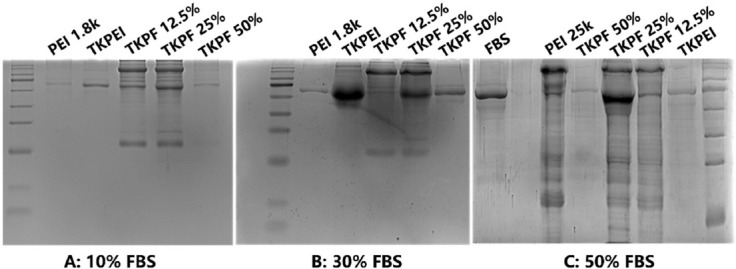
The protein adsorption behavior of TKPEI and TKPFs in DMEM medium containing different concentrations of FBS.

The essential factors mediating serum inhibition effect have been regarded as the large amount of adsorbed protein to form protein corona on the surface of the nanocomposite [30]. Protein corona can affect nanocomposites by causing structure collapse and content leakage. This can hinder the interaction of nanocomposites with cells by changing their physical and chemical properties, which compromises the transfection capacity of gene vectors [31]. Therefore, we investigated the types and amount of absorbed protein on polyplexes to analyze the impact on transfection. The results in Figure 6 showed that the protein adsorption was consistent with the transfection performance of vectors. The gene vectors (PEI25K, PF12.5%, and PF25%) that exhibited poorer transfection efficiency in DMEM containing FBS adsorbed more protein. Specifically, PEI25K absorbed various and large amounts of protein, which partly explained its typical serum inhibitory characteristics. On the contrary, only one kind of protein (with the most albumin in the serum) was absorbed by both TKPEI and TKPF 50%, and the adsorption amount is small, indicating that the interaction of TKPEI and TKPF 50% with serum proteins was weak (Figure 6). The protein adsorption behavior of TKPF 12.5% was similar to that of TKPF 25% and in particular, TKPF 25% showed the most types and the largest amount of adsorbed protein, which was consistent with the worst transfection performance. Hence, reducing the adsorbed protein is a practical method to keep vectors stable in serum and maintain transfection ability.

### Study on cellular uptake

**FIGURE 7:**
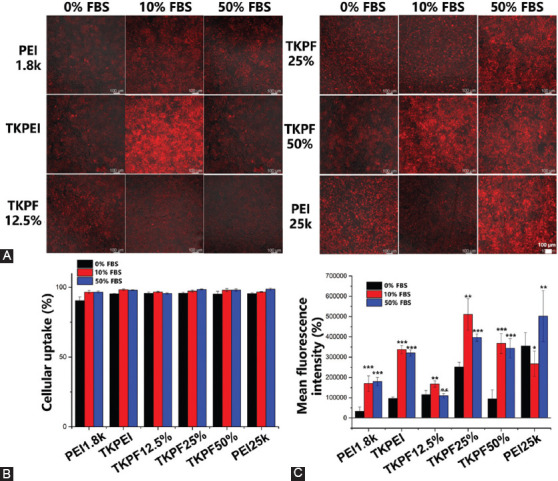
Cellular uptake of Cy5-labelled pDNA in 293TN cells after treatment with TKPEI and TKPFs polyplexes in the presence of 0%, 10% or 50% FBS for 6h. (A) Fluorescence microscopy images; (B) Percentage of Cy5-positive cell; (C) Fluorescence intensity. (Scale bar: 100 µm).

**FIGURE S3:**
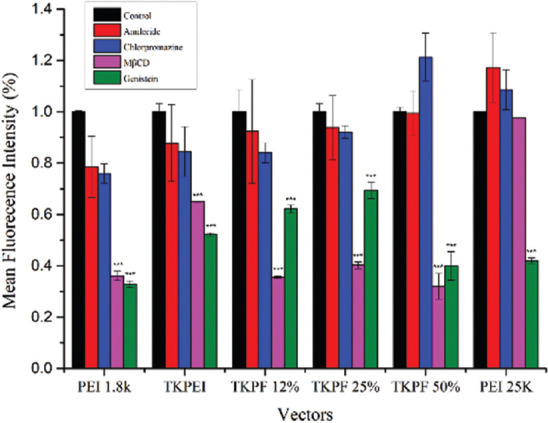
Cellular uptake of TKPF/pDNA polyplexes with the addition of different inhibitors by flow cytometry analysis.

Efficient cellular uptake is a crucial step for achieving high transfection efficiency [32]. 293TN cells were transfected by the synthesized gene vectors encapsulating Cy5-labelled pDNA for 6 h. The results in Figure 7B showed that the uptake efficiency for all gene vectors reached almost 95% without significant difference. In Figure 7C, the constructed carriers basically showed a tendency to promote the uptake of Cy5 in low concentration of FBS (10%), and to inhibit the uptake in high concentrations of FBS (50%). On the one hand, all gene vectors in 10% FBS promoted the cellular uptake. These results might be credited to the small amount of albumin adsorption, which facilitated the transfection polyplexes to enter the cell through the albumin receptor. On the other hand, high concentration of FBS significantly reduced the amount of Cy5 taken up by a single cell for TKPEI, which accounted for the limited transfection performance. TKPF 50% maintained a high cellular uptake amount in 50% FBS, which was also consistent with the result of serum transfection. Although the transfection efficiency of PEI25K was very low in 50% FBS, the uptake amount was extremely high, indicating that the influence of serum on transfection efficiency for PEI25K did not relate to the cellular uptake process. Cellular uptake mechanism was also assessed by addition of uptake blocking reagent. The results in Figure S3 showed that the cellular uptake of PEI1.8K, TKPEI and PEI25K was both inhibited by GNT and m-βCD, indicating caveolae-mediated and lipid raft-mediated endocytosis. The uptake of fluorinated carriers was dominated by lipid rafts, suggesting that the participation of fluorine would alter the mechanism of cellular uptake.

### Lysosomal escape

**FIGURE 8:**
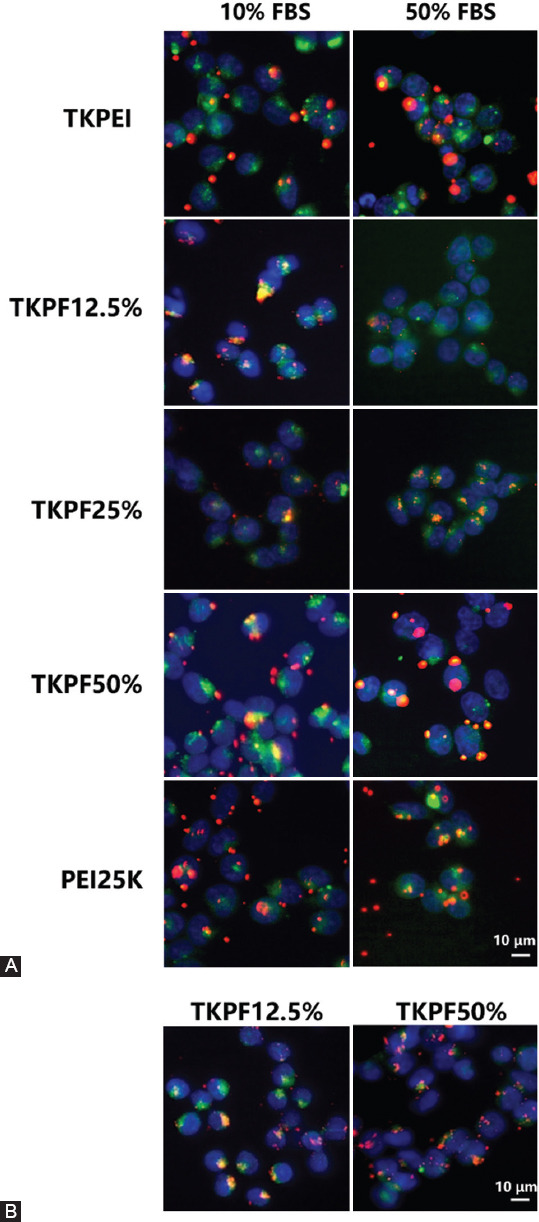
Intracellular distribution of Cy5-labelled pDNA (red) polyplexes at optimal transfection weight ratio for 6 h (A) in the presence of 10% and 50% FBS and 4 hours (B) in 50% FBS. The nuclei were stained with Hoechest 33342 (blue), and the endosome/lysosomes were stained with Lysotracker Green (green). (Scale bar: 10 µm).

Escape from lysosomes is another significant step required for achieving high transfection efficiency [33]. The co-localization of endosomes/lysosomes, nuclei, and genes directly showed the condition of lysosomal escape of different vectors at different FBS concentrations at 6 hours. As shown in Figure 8, both TKPEI and TKPF 50% exhibited effective lysosome escape at 6 hours. And fewer genes in TKPF 12.5% treatment group with 50% FBS entered the cell and almost none escaped from the lysosome. At 4 hours, most of the TKPF 50% fluorescence separated from the Lysotracker signal, while TKPF 12.5% was still trapped in the lysosome. TKPF 25% group was difficult to escape lysosome in high serum concentration despite many Cy5-labelled genes entered the cell, resulting in low transfection. PEI25K was more typical in effective cellular uptake and failure in lysosomal escape. In summary, the results showed that the lysosomal escape efficiency was easily affected by the protein corona of the nanocomposite, and the formed nanocomposite wrapped in the protein corona was more likely to be trapped in the lysosome and then degraded and cleared.

## DISCUSSION

Classic cationic polymer PEI is generally criticized owing to the relatively low transfection efficacy and severe cytotoxicity. Although PEI has been modified with peptides [34], amino acids [35], saccharides, [36] and lipids [37] to promote the transfection performance, the overall transfection properties still require improvement [38]. On one hand, introducing stimuli-responsiveness in crosslinked low molecular weight PEI are promising in order to improve safety issues and enhance DNA release capacity and subsequent transfection. For potential application such as cancer, ROS-sensitive linker can be exploited owing to the high ROS level in tumors, among which thioether are typical with direct cleavage ability [39]. On the other hand, fluorinated chains grafting has been proven effective to endow the polycations with good serum stability owing to the hydrophobicity and lipophobicity, and enhance cellular uptake and endosomal escape due to the affinity to the membrane [24]. Hence, we propose a combination of crosslinking strategies and fluorination techniques to construct gene carriers with excellent transfection efficiency and reduced cytotoxicity. In addition, we wish to analyze the size, surface charge, pDNA loading efficiency, serum tolerance, cytotoxicity, transfection efficiency, cellular uptake, and lysosome escape of the polycation/pDNA polyplexes. In this work, different fluorination ratios of crosslinking gene vectors (PF12.5%, PF25%, and PF50%) were fabricated through crosslinking PEI1.8K with TK linker and employing surface fluorination technology. Among them, TKPF 50% revealed the best transfection performance and serum resistant capacity, which attained high transfection efficiency in the presence of high concentration of FBS. The unique serum resistance of fluorinated polymers is attributed to the hydrophobic property to resist adsorption of serum proteins on the surfaces of polyplexes and to prevent nonspecific interaction with serum proteins [40]. The results of cytotoxicity evaluation and in vitro transfection showed that the introducing fluorine atom was not always beneficial because TKPF 12.5% and TKPF 25% were not able to promote transfection and their transfection efficiency were even lower than that of non-fluorinated TKPEI. In addition, simple crosslinking low molecular weight PEI also acquired better anti-serum transfection performance than PEI25K, which means altering space structure might be another effective method to overcome serum inhibition. We further investigated the intracellular kinetics of four synthesized gene vectors including cellular uptake and lysosome escape. TKPEI with the strongest positive surface charge effectively encapsulated negatively charged genes, and it exhibited high cellular uptake and lysosome escape under high or low concentration of serum. TKPF 12.5% and TKPF 25% suffered from poor cellular uptake and insufficient lysosome escape in FBS contained medium, which partly accounted for the relative low transfection efficiency. TKPF 50% revealed high cellular uptake, effective lysosome escape, and potent transfection of plasmids with serum resistance ability, showing huge potential in gene delivery.

## CONCLUSION

Gene vectors with different degree of fluorination were designed and constructed based on PEI1.8K. TKPEI and TKPF 50% exhibited excellent transfection efficiency and anti-serum transfection performance. TKPF 50% maintained nearly 80% transfection efficiency in a 70% FBS environment, showing the promising potential of TKPF 50% as a gene therapy vector in vivo. The nanocomplex of TKPF 50% had a size of about 149 nm and a positive zeta potential of 7.1 ± 0.7 mV. TKPF 50% also revealed neglectable cytotoxicity in concentrations range from 3.125 to 50 μg/mL, which was similar to the result of PEI 1.8K. Only low amount of albumin in the serum was absorbed by TKPF 50%, indicating the weak interaction of TKPF 50% with serum proteins to exhibit serum resistance. The uptake of TKPF 50% dominated by lipid rafts was higher than PEI without fluorination. In addition, TKPF 50% exhibited effective lysosome escape at 6 hours compared to PEI1.8k. Overall, this study provides a strategy to fabricate bio-responsive fluorinated cationic polymers endowing serum resistance with low toxicity and high transfection efficiency.

## References

[1] Carlet M, Völse K, Vergalli J, Becker M, Herold T, Arner A (2021). *In vivo* inducible reverse genetics in patients'tumors to identify individual therapeutic targets. Nature Commun.

[2] Galli MC (2009). Regulatory considerations for translating gene therapy:A European Union perspective. Sci Transl Med.

[3] Friedrich MJ (2009). Gene therapy repair of donor lungs improves outlook for transplantation. JAMA.

[4] Yahya EB, Alqadhi AM (2021). Recent trends in cancer therapy:A review on the current state of gene delivery. Life Sciences.

[5] Roma-Rodrigues C, Rivas-García L, Baptista PV, Fernandes AR (2020). Gene therapy in cancer treatment:Why go nano?. Pharmaceutics.

[6] Ferrari G, Thrasher AJ, Aiuti A (2021). Gene therapy using haematopoietic stem and progenitor cells. Nat Rev Gen.

[7] Evers MM, Miniarikova J, Juhas S, Vallès A, Bohuslavova B, Juhasova J (2018). AAV5-miHTT gene therapy demonstrates broad distribution and strong human mutant huntingtin lowering in a huntington's disease minipig model. Mol Ther.

[8] Bunnell BA, Morgan RA (1998). Gene therapy for infectious diseases. Clin Microbiol Rev.

[9] Jolly D (1994). Viral vector systems for gene therapy. Cancer Gene Ther.

[10] Kawabata K, Takakura Y, Hashida M (1995). The fate of plasmid DNA after intravenous injection in mice:Involvement of scavenger receptors in its hepatic uptake. Pharm Res.

[11] Wu Z, Asokan A, Samulski RJ (2006). Adeno-associated virus serotypes:Vector toolkit for human gene therapy. Mol Ther.

[12] Yin H, Kanasty RL, Eltoukhy AA, Vegas AJ, Dorkin JR, Anderson DG (2014). Non-viral vectors for gene-based therapy. Nat Rev Gen.

[13] Feng N, Liang L, Fan M, Du Y, Chen C, Jiang R (2021). Treating autoimmune inflammatory diseases with an siERN1-nanoprodrug that mediates macrophage polarization and blocks toll-like receptor signaling. ACS Nano.

[14] Yang W, Miyazaki T, Chen P, Hong T, Naito M, Miyahara Y (2021). Block catiomer with flexible cationic segment enhances complexation with siRNA and the delivery performance *in vitro*. Sci Technol Adv Mater.

[15] Li D, Ahmed M, Khan A, Xu L, Walters AA, Ballesteros B (2021). Tailoring the architecture of cationic polymer brush-modified carbon nanotubes for efficient sirna delivery in cancer immunotherapy. ACS Appl Mater Interfaces.

[16] Nam HY, Nam K, Hahn HJ, Kim BH, Lim HJ, Kim HJ (2009). Biodegradable PAMAM ester for enhanced transfection efficiency with low cytotoxicity. Biomaterials.

[17] Lu HH, Huang CH, Shiue TY, Wang FS, Chang KK, Chen Y (2019). Highly efficient gene release in spatiotemporal precision approached by light and pH dual responsive copolymers. Chem Sci.

[18] Patel AK, Kaczmarek JC, Bose S, Kauffman KJ, Mir F, Heartlein MW (2019). Inhaled nanoformulated mRNA polyplexes for protein production in lung epithelium. Adv Mater.

[19] Huang G, Chen Q, Wu W, Wang J, Chu PK, Bai H (2020). Reconstructed chitosan with alkylamine for enhanced gene delivery by promoting endosomal escape. Carbohydrate Polymers.

[20] Lv H, Zhang S, Wang B, Cui S, Yan J (2006). Toxicity of cationic lipids and cationic polymers in gene delivery. J Control Release.

[21] Vaidyanathan S, Anderson KB, Merzel RL, Jacobovitz B, Kaushik MP, Kelly CN (2015). Quantitative measurement of cationic polymer vector and polymer-pDNA polyplex intercalation into the cell plasma membrane. ACS Nano.

[22] Chen G, Wang K, Wang Y, Wu P, Sun M, Oupický D (2018). Fluorination enhances serum stability of bioreducible poly (amido amine) polyplexes and enables efficient intravenous siRNA delivery. Adv Healthc Mater.

[23] Zhang Z, Shen W, Ling J, Yan Y, Hu J, Cheng Y (2018). The fluorination effect of fluoroamphiphiles in cytosolic protein delivery. Nat Commun.

[24] Xiao YP, Zhang J, Liu YH, Zhang JH, Yu QY, Huang Z (2019). Low molecular weight PEI-based fluorinated polymers for efficient gene delivery. Eur J Med Chem.

[25] Jeong GW, Nah JW (2017). Evaluation of disulfide bond-conjugated LMWSC-g-bPEI as non-viral vector for low cytotoxicity and efficient gene delivery. Carbohydrate Polymers.

[26] Fang G, Zeng F, Yu C, Wu S (2014). Low molecular weight PEIs modified by hydrazone-based crosslinker and betaine as improved gene carriers. Colloids Surfaces B Biointerfaces.

[27] Yue C, Yang Y, Zhang C, Alfranca G, Cheng S, Ma L (2016). ROS-responsive mitochondria-targeting blended nanoparticles:Chemo-and photodynamic synergistic therapy for lung cancer with on-demand drug release upon irradiation with a single light source. Theranostics.

[28] Deng R, Yue Y, Jin F, Chen Y, Kung HF, Lin MC (2009). Revisit the complexation of PEI and DNA-how to make low cytotoxic and highly efficient PEI gene transfection non-viral vectors with a controllable chain length and structure?. J Control Release.

[29] Esposito C, Generosi J, Mossa G, Masotti A, Castellano AC (2006). The analysis of serum effects on structure, size and toxicity of DDAB-DOPE and DC-Chol-DOPE lipoplexes contributes to explain their different transfection efficiency. Colloids Surfaces B Biointerfaces.

[30] Zhu D, Yan H, Zhou Z, Tang J, Liu X, Hartmann R (2021). Influence of the modulation of the protein corona on gene expression using polyethylenimine (PEI) polyplexes as delivery vehicle. Adv Healthc Mater.

[31] Francia V, Schiffelers RM, Cullis PR, Witzigmann D (2020). The biomolecular corona of lipid nanoparticles for gene therapy. Bioconjugate Chem.

[32] Figueroa E, Bugga P, Asthana V, Chen AL, Stephen Yan J, Evans ER (2017). A mechanistic investigation exploring the differential transfection efficiencies between the easy-to-transfect SK-BR3 and difficult-to-transfect CT26 cell lines. J Nanobiotechnol.

[33] Shi L, Wu W, Duan Y, Xu L, Xu Y, Hou L (2020). Light-induced self-escape of spherical nucleic acid from endo/lysosome for efficient non-cationic gene delivery. Angew Chem Int Ed Engl.

[34] Hu J, Zhao W, Liu K, Yu Q, Mao Y, Lu Z (2016). Low-molecular weight polyethylenimine modified with pluronic 123 and RGD- or chimeric RGD-NLS peptide:Characteristics and transfection efficacy of their complexes with plasmid DNA. Molecules.

[35] Fu C, Zheng D, Shi H, Tian H, Zhu X, Chen X (2014). Hydrophobic poly (amino acid)-modified PEI-mediated delivery of single-chain antibody scFv1C9 inhibits HepG2 cell cycle process and xenograft growth in nude mice. J Biomater Sci.

[36] Pathak A, Kumar P, Chuttani K, Jain S, Mishra AK, Vyas SP (2009). Gene expression, biodistribution, and pharmacoscintigraphic evaluation of chondroitin sulfate-PEI nanoconstructs mediated tumor gene therapy. ACS Nano.

[37] Wang J, Meng F, Kim BK, Ke X, Yeo Y (2019). *In-vitro* and *in-vivo* difference in gene delivery by lithocholic acid-polyethyleneimine conjugate. Biomaterials.

[38] Pack DW, Hoffman AS, Pun S, Stayton PS (2005). Design and development of polymers for gene delivery. Nat Rev Drug Discov.

[39] Xu Q, He C, Xiao C, Chen X (2016). Reactive oxygen species (ROS) responsive polymers for biomedical applications. Macromol Biosci.

[40] Scanavachi G, Espinosa YR, Yoneda JS, Rial R, Ruso JM, Itri R (2020). Aggregation features of partially unfolded bovine serum albumin modulated by hydrogenated and fluorinated surfactants:Molecular dynamics insights and experimental approaches. J Colloid Interface Sci.

